# Digitizing clinical trials

**DOI:** 10.1038/s41746-020-0302-y

**Published:** 2020-07-31

**Authors:** O. T. Inan, P. Tenaerts, S. A. Prindiville, H. R. Reynolds, D. S. Dizon, K. Cooper-Arnold, M. Turakhia, M. J. Pletcher, K. L. Preston, H. M. Krumholz, B. M. Marlin, K. D. Mandl, P. Klasnja, B. Spring, E. Iturriaga, R. Campo, P. Desvigne-Nickens, Y. Rosenberg, S. R. Steinhubl, R. M. Califf

**Affiliations:** 1 School of Electrical and Computer Engineering, Georgia Institute of Technology, Atlanta, GA 30332 USA; 2 Clinical Trials Transformation Initiative, Duke University, Durham, NC 27708 USA; 3 Coordinating Center for Clinical Trials, Office of the Director, National Cancer Institute at the National Institutes of Health, Bethesda, MD 20892 USA; 4 School of Medicine, New York University, New York, NY 10003 USA; 5 The Lifespan Cancer Institute, Brown University, Providence, RI 02912 USA; 6 National, Heart, Lung and Blood Institute at the National Institutes of Health, Bethesda, MD 20892 USA; 7 VA Palo Alto Health Care System and the Center for Digital Health, Stanford University, Stanford, CA 94305 USA; 8 Department of Epidemiology and Biostatistics, University of California, San Francisco, CA 94143 USA; 9 Intramural Research Program of the National Institute on Drug Abuse at the National Institutes of Health, Baltimore, MD 21224 USA; 10 The Center for Outcomes Research, Yale New Haven Hospital, Yale University, New Haven, CT 06510 USA; 11 Section of Cardiovascular Medicine, Department of Internal Medicine, Yale School of Medicine, New Haven, CT 06510 USA; 12 Department of Health Policy and Management, Yale School of Public Health, New Haven, Connecticut 06510 USA; 13 College of Information and Computer Sciences, University of Massachusetts at Amherst, Amherst, MA 01003 USA; 14 Computational Health Informatics Program at Boston Children’s Hospital, Departments of Biomedical Informatics and Pediatrics, Harvard Medical School, Boston, MA 02115 USA; 15 School of Information, University of Michigan, Ann Arbor, MI 48109 USA; 16 Northwestern University Feinberg School of Medicine, Chicago, IL 60611 USA; 17 National Heart, Lung, and Blood Institute at the National Institutes of Health, Bethesda, MD 20892 USA; 18 Scripps Research Translational Institute, La Jolla, CA 92037 USA; 19 School of Medicine, Duke University, Durham, NC 27710 USA; 20 Verily Life Sciences and Google Health, South San Francisco, CA 94080 USA; 21 Present Address: Fortira at AstraZeneca, Gaithersburg, MD 20877 USA

**Keywords:** Clinical trials, Translational research

## Abstract

Clinical trials are a fundamental tool used to evaluate the efficacy and safety of new drugs and medical devices and other health system interventions. The traditional clinical trials system acts as a quality funnel for the development and implementation of new drugs, devices and health system interventions. The concept of a “digital clinical trial” involves leveraging digital technology to improve participant access, engagement, trial-related measurements, and/or interventions, enable concealed randomized intervention allocation, and has the potential to transform clinical trials and to lower their cost. In April 2019, the US National Institutes of Health (NIH) and the National Science Foundation (NSF) held a workshop bringing together experts in clinical trials, digital technology, and digital analytics to discuss strategies to implement the use of digital technologies in clinical trials while considering potential challenges. This position paper builds on this workshop to describe the current state of the art for digital clinical trials including (1) defining and outlining the composition and elements of digital trials; (2) describing recruitment and retention using digital technology; (3) outlining data collection elements including mobile health, wearable technologies, application programming interfaces (APIs), digital transmission of data, and consideration of regulatory oversight and guidance for data security, privacy, and remotely provided informed consent; (4) elucidating digital analytics and data science approaches leveraging artificial intelligence and machine learning algorithms; and (5) setting future priorities and strategies that should be addressed to successfully harness digital methods and the myriad benefits of such technologies for clinical research.

## Introduction

Clinical trials are required for causal estimation of the efficacy and the safety of new medical treatments, drugs, and devices. However, traditional clinical trials pose challenges that can hinder the efficient conduct of research to develop a knowledge base supporting products for patient communities. Current operational inefficiencies relating to the identification, recruitment, data acquisition, and follow-up of participants inflate costs, increase participant burden, and extend the already-long clinical trial timelines, all of which contribute to low clinical trial participation: for example, only about 8% of cancer patients enroll in cancer trials^1,2^. For people who do not live close to a research site or who have mobility or scheduling constraints, participating can be expensive, or even impossible^3^, thus increasing disparities in access to research and limiting the diversity of participants in a trial.

### What makes a clinical trial “digital”?

**Fig. 1:**
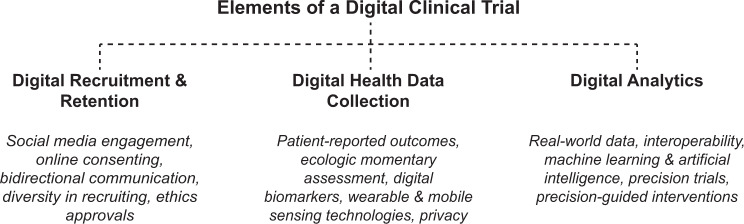
Summary of the key elements of a digital clinical trial. Three elements discussed in this paper include digital recruitment and retention, digital health data collection, and digital analytics.

Enter the concept of *digital clinical trial*, which holds promise as a way to overcome current clinical trial challenges. A digital trial is one that uses technology to improve recruitment and retention, data collection, and analytics (see Fig. 1).

The opportunity to streamline clinical trial costs and efforts through the utilization of digital technologies, while moving more towards a patient-centered trial experience, has captured the interest of the United States (US) National Institutes of Health (NIH) and the National Science Foundation (NSF). The NIH and NSF held a workshop in April 2019 in Bethesda, Maryland bringing together US experts in clinical trials, digital technology, and digital analytics to discuss strategies to implement the use of digital technologies while considering the challenges^4^. In addition, the NIH made pragmatic trials, which include the use of digital technologies, a priority^5^. NIH leaders urge trialists to responsibly adopt digital technologies and other pragmatic features^6^ that disrupt and create efficiencies in a way that preserves the strength of our randomized clinical trials enterprise^7^. The value of digital technologies has also been recognized in two laws passed by Congress that require multiple federal initiatives^8,9^.

There is an important opportunity now to harness digital technology to accelerate the pace at which we generate evidence through clinical trials. Digital technology can improve trial efficiency by enhancing and supporting the role of investigators and study teams. Many trials can be done entirely without in-person visits and in some cases participants might not even meet their study teams. However, trials that that involve serious illnesses, extensive procedures like advanced imaging and biopsies and interventions with significant risk will require close monitoring and oversight by competent clinicians and investigators.

### Rethinking data collection in the digital health era

Digital health technologies already enable user-friendly measurement of participant health markers, including physical activity^10^, sleep^11^, heart rate^12^, medication adherence^13^, and respiration patterns/rate^14^. Many of these technologies have not been developed for research use, but are a part of the burgeoning health and wellness industry. As such, many will need more evaluation and validation before they are fully ready for use in the clinical research setting. For example, some may not have the precision of standard medical devices, but given the longitudinal nature of the data, may produce useful information when evaluated against validated measures.

Despite this, digital health technologies provide the research community with new tools that can greatly enhance the clinical trial enterprise, including the opportunity to run trials liberated from the anchor of “brick and mortar” research centers. A fully digital trial will enable access for potential participants regardless of where they live or work. For investigators, this means more efficient and real-time remote monitoring and multiple opportunities for interactive patient management and assessment (passive and active), which means that more intensive work can be done with budgets that may not require greater resources. This also means investigators can investigate traditionally collected endpoints alongside more novel endpoints—ones that can be captured in an automated fashion and over extended periods. Doing so has the potential to reduce the burden on both research teams and on study participants. Consider the opportunities of pairing geo-location apps on smartphones with electronic health record (EHR) data to identify a study endpoint such as real-time emergency medical appointments and hospitalizations. As such, available and emerging technology represents a potentially transformative approach and evidence for the need for greater efficiency has never been highlighted so well as now, during the time of the COVID-19 pandemic. Indeed, one such randomized trial evaluating a 5-day course of hydroxychloroquine for asymptomatic volunteers following exposure to someone with confirmed infection was launched, enrolled via social and traditional media, consented via electronic means, and all data collected electronically, and published within 90 days^15^. As such, although digital trials are still in their infancy, the ability to more widely operationalize them has taken on new urgency. Recent advances specific to each of these elements are described below in detail.

## Digital recruitment and retention in clinical trials

As discussed above, recruitment, informed consent, and retention of participants is a major impediment to the timely completion of clinical trials. Enrollment rates may be severely curtailed if enrolling a representative population takes priority^16,17^. Often, to meet enrollment milestones, populations for whom services are hard-to-reach are excluded, which may undermine external validity. Multiple reasons exist to help explain this lack of enrollment, including lack of clinician involvement, financial difficulties^18^ and overly restrictive eligibility criteria^19^. The use of digital tools to aid in recruitment, especially for purposes of therapeutic intent trials, is key to more efficient trials, but to move forward, collaboration with the Office of Human Research Protections (OHRP) is critical. While guidance has been issued with respect to the use of new technologies in the informed consent process^20^, similar guidance for recruiting potential study volunteers is lacking. Such guidance is necessary to address the use of social media and other novel communication tools as recruitment tools by local institutional review boards (IRBs), while allowing investigators to react and respond in real-time. For example, requiring review of tweets planned for use in support of a clinical trial might be reasonable; but requiring IRB review of revisions or responses based on real-time feedback may be detrimental to the original intent of why social media outreach was used. Despite these unchartered waters, efforts are being made to help investigators reach potential study volunteers. For example, the SWOG Cancer Research Network proposes the incorporation of a social media toolkit for new clinical trials, aimed at helping investigators and their institutions raise awareness of these studies^21^.

Participant retention may be adversely impacted for other reasons, including concerns regarding randomization and assignment to a placebo arm, lack of understanding related to poorly executed informed consent, lack of renumeration, and a general lack of understanding of the clinical trial process^22^. Digital technologies have the potential to help address all of these issues. Proposed interventions range from tracking applications to allow for more efficient participant outreach, improved opportunities to participate in trials without having to return to the research center, and the use of video and other visual formats to aide informed consent^23^. Where payments are envisioned, efforts to provide payment should maximize use of currently available technologies, bearing in mind the importance of any relevant revenue and taxation limits for study volunteers.

Beyond this, it is widely known that disparities exist even among those who enroll in clinical trials. For example, Black Americans represented only 2.5% and 4% of enrollees in trials involving cardiology or cancer immunotherapy^24,25^. Whether these estimates are accurate is a matter of concern, because almost 85% of clinical trials included in a recent review did not report race/ethnicity data^26^. These findings point to ongoing issues addressing disparities in clinical trials, and potentially another facet that may be addressed through digital technology. Solutions may vary but a potentially actionable solution could focus on the creation of more effective tailored communication strategies to overcome barriers, such as mistrust, access, and fear of human experimentation.

Another demographic group under-represented in clinical trials is older adults. For example, estimates are that 40% of all cardiology clinical trials exclude older adults^27^, despite older age being a well-established factor associated with cardiovascular risk. Similarly, heart failure is primarily a disease affecting the elderly, but those enrolled in clinical trials tend to be much younger than the actual population of patients in that condition^16^. The lack of older adults in trials is especially striking given that older adults are the fastest growing demographic group in many parts of the world and who may be differentially impacted by treatments because of their age^28^. Trials that incorporate remote monitoring to reduce in-person visits could improve research participation for patients who require assistance to get to study sites, including many older patients.

Fortunately, work to harness new technologies to revolutionize the clinical trial enterprise has begun demonstrating that these efforts are feasible. In addition to the randomized trial for volunteers exposed to COVID-19 above, within oncology, the Metastatic Breast Cancer Project^29^ was developed in collaboration with women and men with metastatic breast cancer, physicians, researchers, and advocates. The project harnesses engagement through social media and addresses the barrier to recruitment and retention through online consent and bidirectional communication that supports information sharing throughout the trial timeframe. As reported in 2017, almost 3000 volunteers signed up for the registry in its first year with 95% completing the required survey about their cancer, treatments, and demographics^29^.

In addition, recent work supported by the National Library of Medicine and ClinicalTrials.gov^30^ has been exploring whether standardized language and machine readable templates can use be used to create a platform for trial recruitment. The templates would be analyzed using artificial intelligence to translate clinical trial requirements and information into common language so that potential practitioners and participants could easily find studies in which they could participate.

## Digital health data collection

Following recruitment of participants for a clinical trial, data must be collected as part of the trial process. Digital data in clinical trials can take many forms, ranging from clinical and demographic data, sensed physiological and activity data, patient reported outcomes and images collected via a smart phone or tablet, electronic medical record data from a vendor API, and biological samples drawn at home or in a local lab^31^. Aside from the biological samples, examples of digital health data collection are described in this section.

### Digital monitoring and biomarkers

The term *digital* biomarker typically refers specifically to objective measures of physiologic, pathologic, anatomic, behavioral, social or activity characteristics, and patient self-report that can be acquired using digital technology and “evaluated as an indicator of normal biologic processes, pathologic processes, or biological responses to therapeutic interventions”^32,33^. While many digital biomarkers are still being validated, over time they may provide in-depth information regarding physiological processes central to informing diagnostics, dosing titration and as endpoints for clinical trials. Examples include wearable sweat sensing for glucose and lactate, as well as electrolytes^34^, cardiogenic chest wall vibrations to assess clinical status of patients with heart failure^35^, or structural health markers for knee joint injury obtained through a brace^36^.

Digital technologies can also be leveraged to enable the collection of data that could not be obtained previously. For example, Zhan et al. required subjects to perform five cognitive tasks on a smartphone application, then merged the task data to form a validated mobile Parkinson’s disease score^37^. Brogioli and colleagues validated wearable sensors to generate a neurological classification in spinal cord injury^38^. Recently Food and Drug Administration (FDA) approved digital approaches to detect rhythm abnormalities, such as atrial fibrillation, through sensors in the Apple Watch, including electrodes for electrocardiography and optical sensors for photoplethysmography^39,40^.

### Safety monitoring in digital clinical trials

As digital technology finds its way in to routine clinical trial processes, researchers are increasingly pairing new tools with traditional biomarker assessments as a way to validate their safety and usability^32^. Harnessing the ability for digital tools to collect data continuously, which can be transmitted directly to researchers, might improve the detection of infrequent events or those that are situation-specific and unlikely to occur during a study visit. The speed at which adverse and safety events can be identified and reported may have a significant impact on the timeliness of completion and reporting of clinical trials.

Nevertheless, critical issues remain that will impact the implementation of digital tools, including their evaluation to ensure they meet standards for reliability and validity, which can be harder to establish for detection of fleeting, momentary or novel events. As important, the expectations on study participants, investigators, and trial sponsors need to be better defined. Attempts are being made to do just this. For example, collaborative recommendations are provided by the Clinical Trials Transformation Initiative (CTTI) for dealing with data collection from mobile technologies, as summarized in Table 1^41^.

**Table 1:** Clinical trials transformation initiative (CTTI) recommendations for dealing with data collection from mobile technologies (Adapted from ref. ^41^).

1		Design the mobile technologies using evidence-based principles. Address clearly user-centered design principles in developing and choosing technology. Proactively address data privacy and security with user input.
2		Collect the appropriate dataset necessary to address the study aims:
	a	Evidence-based principles should drive decisions about the quantity of data to be collected,
	b	Ensure that appropriate metadata are collected to provide sufficient contextual information to understand the data captured by mobile technologies while minimizing the collection of intrusive data, and
	c	The most appropriate measurement intervals and optimal sampling frequency for a given outcome should be determined during development of the study aims.
3		Optimize data collection. When using mobile technologies for data capture, a multi-pronged approach to optimize data quality and missing is necessary, with efforts focused on:
	a	Optimizing trial design,
	b	Including appropriate strategies for monitoring and optimizing data quality,
	c	Ensuring technical approaches are in place to address technology-related or transmission-related causes of missing data,
	d	Identifying acceptable ranges and mitigate variability in data collected via mobile technologies, and
	e	Piloting testing to identify any unanticipated causes of missing data.
4		Proactively plan for the analysis of data captured using mobile technologies.
5		Establish common metrics, norms and/or standards to drive the successful scaling and more rapid acceptance of mobile technologies for data capture.

### Data security (privacy and security)

It is clear that digital health technology has created challenges in the efforts to modernize standards for privacy, safety, ethics and regulation. Digital technology and remote monitoring heighten the need for security measures to protect against data breaches during collection, transmission, and/or storage of data. While identifiable patient data require protection, some measures, such as GPS/location data^42^, could put trial participants at particular risk for legal actions and economic loss due to stigma^41^. To this end, the FDA has embraced cybersecurity as a component of medical device certification^43^. Even as we strive to increase patient access, the risks and benefits of sharing individual and/or pooled patient data must be conveyed to all stakeholders. Breach of confidential databases remains a risk, although the use of distributed ledgers, such as blockchain, or decentralized databases, could mitigate risk.

Digital trials may benefit from guidance for IRBs, especially as it pertains to consenting requirements, reporting, and oversight. For example, a site-less study may include virtual visits across state lines or national borders rather than at a specific physical study site, and differences in state regulations pertaining to investigator oversight must be considered in order for trials to effectively operate. In the absence of explicit cross-state telehealth licensing agreements, an Interstate Medical Licensure Compact may address this issue, and encouraging all states to participate may be critical^44^. Further, options for documentation of informed consent will need to incorporate remote consent technologies, while maintaining certainty that consent covers data collection, transmission, sharing, and security.

## Data analytics

### Real-world data

The digital transformation of healthcare data presents marked opportunities to improve clinical trials, from trial matching to data collection, using real world data collected in EHRs^45^, medical devices, and technology in Internet of Things, as well as other sources. Truly flexible, adaptable, extensible, and scalable clinical trial infrastructure that utilizes the EHR is now possible; the health record is converging on a few forms of interoperability that will underpin an apps-driven information economy^46^. For example, the SMART (Substitutable Medical Applications, Reusable Technologies) on FHIR (Fast Healthcare Interoperability Resources) API enables medical researchers, clinical providers, and patients to connect apps to the health system across EHR platforms^47^. As part of such an automated real-world data system, data from sensors, mobile devices, patient-generated data and patient-reported outcomes could become more routine trial markers and endpoints^48^. The SMART/HL7 Flat FHIR/Bulk Data Export API^49^ provides a standardized approach to readily create population-level datasets from EHR. A principal challenge will be data quality, which is currently being addressed throughout the research ecosystem^50^, including by the FDA. For example, data mapping, which focuses attention on the standardization and validation of data is being explored, and efforts to create common data models are also addressing this challenge^51,52^ as are emerging technologies to automate processes.

### Advanced analytics

Machine learning and artificial intelligence enables the development of advanced analytic methods that can be brought to bear on multiple facets of the conduct of digital clinical trials. For example, automatically inferred clusters or sub-types can be used to bootstrap the personalization of supervised detection methods, providing greater detection accuracy. Similarly, supervised and unsupervised learning methods can be used to passively detect context and infer proximal outcomes while leveraging reinforcement learning methods to optimize intervention component selections based on the inferred context. These approaches can be used to match participants to studies, to improve digital data extraction and computational phenotyping, and to augment efforts to interpret the trial findings.

### Optimizing trial methods

Digital approaches also lend themselves to a range of methods, such as micro-randomization, to optimize and personalize clinical trial methods^53–55^. Micro-randomized trials^56,57^, a method that repeatedly randomizes provision of an intervention to each person each time that intervention may be provided, can identify factors such as timing and dose for optimally delivering digital trial tools such as reminders and engagement strategies for recruiting, enrolling, and retaining trail participants. In addition, such methods can be used to personalize the delivery of such trial tools to maximize their effectiveness for each participant^58^. Since randomizations intended to optimize trial technologies are often decoupled from the randomizations used to investigate the primary trial question, in many cases both the health and trial technology optimization questions can be investigated concurrently. Over time, such optimization experiments will result in a robust evidence base about how to most effectively recruit, consent, and engage participants in digital trials, greatly enhancing this important innovation in the conduct of biomedical research.

## Strategies for the future

The current methods for conducting clinical trials are not sustainable, and will leave a chasm between the need for evidence to inform health and healthcare and the availability of that evidence. New strategies for the future of clinical trials are needed. Suggested strategies for disruptive clinical trials that were suggested by participants in the NIH/NSF Workshop are summarized in Table 2^4^.

**Table 2:** Research opportunities and potential action Items from the NIH/NSF workshop on digital clinical trials.

Research opportunity	Potential action Item
Leveraging digital technologies to transform clinical trials	• Capitalize on existing technologies and research platforms
	• Incorporate testing and the adoption of new approaches
	• Leverage the science from which other areas of information technology have already benefited (e.g., Artificial Intelligence)
	• Ensure trial technology tools are accessible for people with low digital and reading literacy
	• Develop partnerships with new technology and computational communities
Incorporating technology to enhance clinical trials	• Develop standard protocol templates that include automation for recruitment, retention, and data collection
	• Develop validation models for new devices and analyses using existing trials and tools
	• Develop common standards for data collection and transmission and the use of standard data elements
	• Create depositories of digital tools and methods
Committing to use of digital technology to address disparities	• Develop partnerships between technology developers, researchers and community advocates
	• Make investments in the next generation workforce in medicine, technology, and clinical research
	• Bring broadband and Wi-Fi access to rural communities

## Conclusions

The creation of a digital clinical trial enterprise in the US will require empirical research on the risks and benefits of digital trials. This also includes research addressing the privacy and security concerns, training protocols, and potential negative impact of using technology. Efforts should also target development of an evidence base of effective intervention components that can readily inform future intervention development. Implementation will also require research that is higher in risk than past clinical trials as there is a steep learning curve for digital technologies in the biomedical space. Most of those that now develop these technologies are not partnered with clinical researchers, while clinical researchers do not have the skills that support digital clinical trials. To develop the knowledge needed to spur a robust digital clinical trial enterprise will require substantial commitment amongst leadership facing initial challenges, and is required for growth.

The clinical trial research team of the future will have a different composition compared with those of today. Research teams will be transformed to include computer scientists and engineers as critical partners for both technology development, but also data collection, analysis, security, and privacy. These new teams will also have increased technological and data science knowledge among all involved with conducting trials, so that they have a common language with their technical partners and know which questions they should be asking. This includes investigators, research team, clinical staff, regulatory officials, which are all necessary to successfully develop and implement digital trials^41^. In the future, simply purchasing app development from a third party vendor will not be enough to sustain a digital clinical trial. Diverse partnerships within the team must go deeper to develop tools that engage and sustain participants safely and effectively.

Education on digital technology for IRBs should continue. This builds off the work of the Public Responsibility in Medicine and Research group that has been providing training in digital technologies for health for almost a decade. In addition, members of data safety and monitoring boards (DSMBs) should also be trained on the safe and effective use of technologies, including the risks that technology introduces into the system. Teams should also be informed of the state-of-the-art work being done by FDA and National Institute of Standards and Technology (NIST) on cybersecurity and privacy in medical settings and other best practices.

In summary, the traditional clinical trials system cannot keep pace with the need to evaluate new drugs, devices, and health system interventions or to conduct much needed comparative effectiveness research. Over the last two decades, people have been increasingly embracing innovative solutions built around the reach, convenience and value digital technologies bring them. Many interactions in the past that required face-to-face contact with specific professionals, such as a travel agent or bank teller, are now routinely carried out by individuals in their home on their computer or smartphone. Although the health care and clinical trials enterprises have been slower to adopt digital alternatives to traditional systems, recently, a range of digital tools that can enable the reengineering of health care and clinical research have become available and continue to expand rapidly. Key components, such as medical grade personal sensors, individual access and control of health records, and most challenging, personalized digital communications, are already being incorporated in clinical research, although still most commonly in small pilot programs. Many challenges remain and lessons will be learned as digital research is moved into the mainstream, but knowledge of the innumerable benefits to clinical research reinforces the view that now is the time to support digital methods with a focus on learning the most effective and efficient methods.
